# Supplementary material for: The physiological response of Ectomycorrhizal fungus Lepista sordida to Cd and Cu stress
Source: PeerJ. 2021 Apr 16;9:e11115. doi: 10.7717/peerj.11115 (PMC8054734; doi:10.7717/peerj.11115)
Supplement: Supplemental Information 1 [file peerj-09-11115-s001.pdf]

BLAST ® » blastn suite » RID-EGABFX59016

BLAST Results

Questions/comments

Job title: Z864-HLXM

**RID**	EGABFX59016 (Expires on 06-17 10:07 am)
**Query ID**	lcl|Query_49443
**Description**	None
**Molecule type**	dna
**Query Length**	643
**Database Name**	nt
**Description**	Nucleotide collection (nt)
**Program**	BLASTN 2.10.1+

Graphic Summary

Distribution of the top 100 Blast Hits on 100 subject sequences

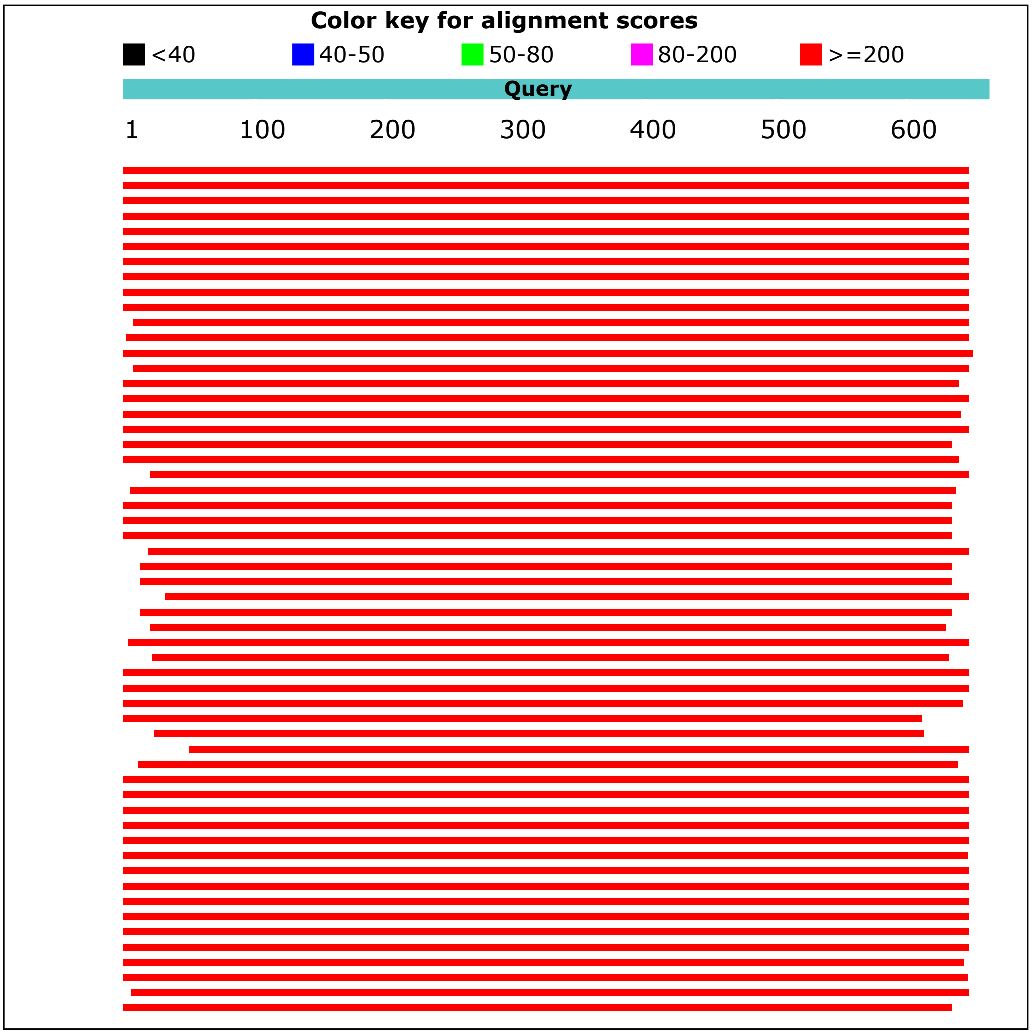

Descriptions

Sequences producing significant alignments:

Description	Max Score	Total Score	Query Cover	E value	Per. Ident	Accession
Lepista sordida culture-collection MFLUCC:13-0898 18S ribosomal RNA gene, partial sequence; internal transcribed spacer 1, 5.8S ribosomal RNA gene, and internal transcribed spacer 2, complete sequence; and 28S ribosomal RNA gene, partial sequence	1173	1173	99%	0.0	99.69%	KU877531.1
Lepista sordida culture-collection MFLUCC:14-0769 18S ribosomal RNA gene, partial sequence; internal transcribed spacer 1, 5.8S ribosomal RNA gene, and internal transcribed spacer 2, complete sequence; and 28S ribosomal RNA gene, partial sequence	1173	1173	99%	0.0	99.69%	KU877530.1
Lepista sordida culture-collection MFLUCC:12-0476 18S ribosomal RNA gene, partial sequence; internal transcribed spacer 1, 5.8S ribosomal RNA gene, and internal transcribed spacer 2, complete sequence; and 28S ribosomal RNA gene, partial sequence	1173	1173	99%	0.0	99.69%	KU877529.1
Lepista sordida voucher TNS-F-61698 18S ribosomal RNA gene, partial sequence; internal transcribed spacer 1, 5.8S ribosomal RNA gene, and internal transcribed spacer 2, complete sequence; and 28S ribosomal RNA gene, partial sequence	1173	1173	99%	0.0	99.69%	KP293583.1
Lepista sordida genomic DNA sequence contains ITS1, 5.8S rRNA gene, ITS2, strain EGDA2	1173	1173	99%	0.0	99.69%	LN827702.1
Lepista sordida strain qqhe1 18S ribosomal RNA gene, partial sequence; internal transcribed spacer 1, 5.8S ribosomal RNA gene, and internal transcribed spacer 2, complete sequence; and 28S ribosomal RNA gene, partial sequence	1173	1173	99%	0.0	99.69%	KF874612.1
Lepista sordida isolate SMCC39.01.1 18S ribosomal RNA gene, partial sequence; internal transcribed spacer 1, 5.8S ribosomal RNA gene, and internal transcribed spacer 2, complete sequence; and 28S ribosomal RNA gene, partial sequence	1173	1173	99%	0.0	99.69%	FJ501562.1
Lepista sordida small subunit ribosomal RNA gene, partial sequence; internal transcribed spacer 1, 5.8S ribosomal RNA gene, and internal transcribed spacer 2, complete sequence; and large subunit ribosomal RNA gene, partial sequence	1170	1170	99%	0.0	99.53%	KJ137272.1
Lepista sordida isolate SMCC39.01.4 18S ribosomal RNA gene, partial sequence; internal transcribed spacer 1, 5.8S ribosomal RNA gene, and internal transcribed spacer 2, complete sequence; and 28S ribosomal RNA gene, partial sequence	1168	1168	99%	0.0	99.53%	FJ501563.1
Lepista sordida strain IFO 31013 small subunit ribosomal RNA gene, partial sequence; internal transcribed spacer 1, 5.8S ribosomal RNA gene and internal transcribed spacer 2, complete sequence; and large subunit ribosomal RNA gene, partial sequence	1168	1168	99%	0.0	99.53%	AF241523.1

Description	Max Score	Total Score	Query Cover	E value	Per. Ident	Accession
Lepista sordida voucher KA17-0551 small subunit ribosomal RNA gene, partial sequence; internal transcribed spacer 1 and 5.8S ribosomal RNA gene, complete sequence; and internal transcribed spacer 2, partial sequence	1164	1164	98%	0.0	99.84%	MN294842.1
Lepista sordida small subunit ribosomal RNA gene, partial sequence; internal transcribed spacer 1, 5.8S ribosomal RNA gene, and internal transcribed spacer 2, complete sequence; and large subunit ribosomal RNA gene, partial sequence	1162	1162	99%	0.0	99.53%	MH234513.1
Lepista sordida voucher EL4-03 (GB) 18S ribosomal RNA gene, partial sequence; internal transcribed spacer 1, 5.8S ribosomal RNA gene, and internal transcribed spacer 2, complete sequence; and 28S ribosomal RNA gene, partial sequence	1160	1160	100%	0.0	99.22%	JN649350.1
Lepista sordida 18S ribosomal RNA gene, partial sequence; internal transcribed spacer 1, 5.8S ribosomal RNA gene, and internal transcribed spacer 2, complete sequence; and 28S ribosomal RNA gene, partial sequence	1158	1158	98%	0.0	99.68%	FJ428582.1
Lepista sordida strain MKACC 50097 internal transcribed spacer 1, partial sequence; 5.8S ribosomal RNA gene, complete sequence; and internal transcribed spacer 2, partial sequence	1157	1157	98%	0.0	99.68%	AY534114.1
Lepista tarda isolate 4517 internal transcribed spacer 1, partial sequence; 5.8S ribosomal RNA gene and internal transcribed spacer 2, complete sequence; and 28S ribosomal RNA gene, partial sequence	1155	1155	99%	0.0	99.22%	KM406961.1
Lepista sordida voucher A22 internal transcribed spacer 1, partial sequence; 5.8S ribosomal RNA gene, complete sequence; and internal transcribed spacer 2, partial sequence	1155	1155	98%	0.0	99.53%	JX434648.1
Lepista sp. BAB-4764 18S ribosomal RNA gene, partial sequence; internal transcribed spacer 1, 5.8S ribosomal RNA gene, and internal transcribed spacer 2, complete sequence; and 28S ribosomal RNA gene, partial sequence	1151	1151	99%	0.0	99.07%	KR154993.1
Lepista sordida voucher FLAS-F-60023 internal transcribed spacer 1, partial sequence; 5.8S ribosomal RNA gene and internal transcribed spacer 2, complete sequence; and large subunit ribosomal RNA gene, partial sequence	1144	1144	97%	0.0	99.52%	KY654743.1
Lepista sordida strain CBS 341.69 18S ribosomal RNA gene, partial sequence; internal transcribed spacer 1, 5.8S ribosomal RNA gene, and internal transcribed spacer 2, complete sequence; and 28S ribosomal RNA gene, partial sequence	1142	1142	98%	0.0	99.21%	FJ770391.1
Lepista sordida isolate ALV7992 small subunit ribosomal RNA gene, partial sequence; internal transcribed spacer 1 and 5.8S ribosomal RNA gene, complete sequence; and internal transcribed spacer 2, partial sequence	1140	1140	96%	0.0	99.84%	MH644795.1
Lepista sordida voucher FLAS-F-61662 internal transcribed spacer 1, partial sequence; 5.8S ribosomal RNA gene, complete sequence; and internal transcribed spacer 2, partial sequence	1140	1140	97%	0.0	99.52%	MH212036.1

Description	Max Score	Total Score	Query Cover	E value	Per. Ident	Accession
Lepista sordida isolate LMBC 171 internal transcribed spacer 1, partial sequence; 5.8S ribosomal RNA gene and internal transcribed spacer 2, complete sequence; and large subunit ribosomal RNA gene, partial sequence	1134	1134	97%	0.0	99.36%	MN625448.1
Lepista sordida voucher ubc F32595 18S ribosomal RNA gene, partial sequence; internal transcribed spacer 1, 5.8S ribosomal RNA gene, and internal transcribed spacer 2, complete sequence; and 28S ribosomal RNA gene, partial sequence	1131	1131	97%	0.0	99.20%	MG969982.1
Lepista sordida voucher ubc F32589 18S ribosomal RNA gene, partial sequence; internal transcribed spacer 1, 5.8S ribosomal RNA gene, and internal transcribed spacer 2, complete sequence; and 28S ribosomal RNA gene, partial sequence	1131	1131	97%	0.0	99.20%	MG969981.1
Lepista sordida voucher 201901100801 small subunit ribosomal RNA gene, partial sequence; internal transcribed spacer 1 and 5.8S ribosomal RNA gene, complete sequence; and internal transcribed spacer 2, partial sequence	1129	1129	96%	0.0	99.36%	MN998150.1
Lepista sordida voucher SYAU-FUNGI-040 internal transcribed spacer 1, partial sequence; 5.8S ribosomal RNA gene and internal transcribed spacer 2, complete sequence; and 28S ribosomal RNA gene, partial sequence	1123	1123	95%	0.0	99.67%	MK116606.1
Lepista sordida voucher SYAU-FUNGI-039 internal transcribed spacer 1, partial sequence; 5.8S ribosomal RNA gene and internal transcribed spacer 2, complete sequence; and 28S ribosomal RNA gene, partial sequence	1123	1123	95%	0.0	99.67%	MK116605.1
Lepista sordida voucher GIM5.315 18S ribosomal RNA gene, partial sequence; internal transcribed spacer 1 and 5.8S ribosomal RNA gene, complete sequence; and internal transcribed spacer, partial sequence	1120	1120	94%	0.0	99.84%	JF420892.1
Lepista sordida voucher SYAU-FUNGI-044 internal transcribed spacer 1, partial sequence; 5.8S ribosomal RNA gene and internal transcribed spacer 2, complete sequence; and 28S ribosomal RNA gene, partial sequence	1118	1118	95%	0.0	99.51%	MK116608.1
Clitocybe tarda var. alcalina voucher TENN:023387 internal transcribed spacer 1, partial sequence; 5.8S ribosomal RNA gene, complete sequence; and internal transcribed spacer 2, partial sequence	1107	1107	93%	0.0	99.83%	HQ179666.1
Lepista sordida voucher HFJAU0263 small subunit ribosomal RNA gene, partial sequence; internal transcribed spacer 1 and 5.8S ribosomal RNA gene, complete sequence; and internal transcribed spacer 2, partial sequence	1099	1099	99%	0.0	97.80%	MN258660.1
Lepista sp. voucher MES-1615 internal transcribed spacer 1, partial sequence; 5.8S ribosomal RNA gene, complete sequence; and internal transcribed spacer 2, partial sequence	1098	1098	93%	0.0	99.50%	KY462501.1

Description	Max Score	Total Score	Query Cover	E value	Per. Ident	Accession
Lepista nuda voucher 420526MF0004 small subunit ribosomal RNA gene, partial sequence; internal transcribed spacer 1, 5.8S ribosomal RNA gene, and internal transcribed spacer 2, complete sequence; and large subunit ribosomal RNA gene, partial sequence	1096	1096	99%	0.0	97.51%	MG719766.1
Lepista sordida strain xsd08104 18S ribosomal RNA gene, partial sequence; internal transcribed spacer 1, 5.8S ribosomal RNA gene, and internal transcribed spacer 2, complete sequence; and 28S ribosomal RNA gene, partial sequence	1096	1096	99%	0.0	97.51%	FJ481015.1
Lepista sordida strain ts916 internal transcribed spacer 1, partial sequence; 5.8S ribosomal RNA gene and internal transcribed spacer 2, complete sequence; and large subunit ribosomal RNA gene, partial sequence	1090	1090	98%	0.0	97.64%	MG516585.1
Lepista sordida voucher G1 internal transcribed spacer 1, partial sequence; 5.8S ribosomal RNA gene and internal transcribed spacer 2, complete sequence; and large subunit ribosomal RNA gene, partial sequence	1081	1081	94%	0.0	98.85%	MF384327.1
Lepista sordida Ls17-1 genes for ITS1, 5.8S rRNA, ITS2, 28S rRNA, partial and complete sequence	1072	1072	90%	0.0	99.83%	LC370449.1
Lepista sordida var. aianthina strain MCVE 28714 small subunit ribosomal RNA gene, partial sequence; internal transcribed spacer 1 and 5.8S ribosomal RNA gene, complete sequence; and internal transcribed spacer 2, partial sequence	1070	1070	91%	0.0	99.32%	KU577457.1
Lepista sordida voucher MHHNU 31387 internal transcribed spacer 1, partial sequence; 5.8S ribosomal RNA gene, complete sequence; and internal transcribed spacer 2, partial sequence	1051	1051	96%	0.0	97.26%	MK214400.1
Clitocybe cf. nuda voucher JLF8423 small subunit ribosomal RNA gene, partial sequence; internal transcribed spacer 1, 5.8S ribosomal RNA gene, and internal transcribed spacer 2, complete sequence; and large subunit ribosomal RNA gene, partial sequence	1037	1037	99%	0.0	95.81%	MT360912.1
Tricholoma mongolicum voucher HMJAU:24946 18S ribosomal RNA gene, partial sequence; internal transcribed spacer 1, 5.8S ribosomal RNA gene, and internal transcribed spacer 2, complete sequence; and 28S ribosomal RNA gene, partial sequence	1037	1037	99%	0.0	95.95%	KC413949.1
Tricholoma mongolicum voucher HMJAU:24945 18S ribosomal RNA gene, partial sequence; internal transcribed spacer 1, 5.8S ribosomal RNA gene, and internal transcribed spacer 2, complete sequence; and 28S ribosomal RNA gene, partial sequence	1037	1037	99%	0.0	95.95%	KC413948.1
Tricholoma mongolicum voucher HMJAU:24940 18S ribosomal RNA gene, partial sequence; internal transcribed spacer 1, 5.8S ribosomal RNA gene, and internal transcribed spacer 2, complete sequence; and 28S ribosomal RNA gene, partial sequence	1037	1037	99%	0.0	95.95%	KC413943.1

Description	Max Score	Total Score	Query Cover	E value	Per. Ident	Accession
Lepista saeva voucher 3684 small subunit ribosomal RNA gene, partial sequence; internal transcribed spacer 1, 5.8S ribosomal RNA gene, and internal transcribed spacer 2, complete sequence; and large subunit ribosomal RNA gene, partial sequence	1033	1033	99%	0.0	95.81%	MH930154.1
Tricholoma mongolicum voucher HMJAU:24939 18S ribosomal RNA gene, partial sequence; internal transcribed spacer 1, 5.8S ribosomal RNA gene, and internal transcribed spacer 2, complete sequence; and 28S ribosomal RNA gene, partial sequence	1033	1033	99%	0.0	95.94%	KC413942.1
Tricholoma mongolicum voucher HMJAU:24942 18S ribosomal RNA gene, partial sequence; internal transcribed spacer 1, 5.8S ribosomal RNA gene, and internal transcribed spacer 2, complete sequence; and 28S ribosomal RNA gene, partial sequence	1029	1029	99%	0.0	95.79%	KC413945.1
Tricholoma mongolicum voucher HMJAU:24941 18S ribosomal RNA gene, partial sequence; internal transcribed spacer 1, 5.8S ribosomal RNA gene, and internal transcribed spacer 2, complete sequence; and 28S ribosomal RNA gene, partial sequence	1029	1029	99%	0.0	95.79%	KC413944.1
Uncultured fungus clone 4248_938 small subunit ribosomal RNA gene, partial sequence; internal transcribed spacer 1, 5.8S ribosomal RNA gene, and internal transcribed spacer 2, complete sequence; and large subunit ribosomal RNA gene, partial sequence	1026	1026	99%	0.0	95.65%	MT236893.1
Leucocalocybe mongolica strain ba small subunit ribosomal RNA gene, partial sequence; internal transcribed spacer 1, 5.8S ribosomal RNA gene, and internal transcribed spacer 2, complete sequence; and large subunit ribosomal RNA gene, partial sequence	1026	1026	99%	0.0	95.65%	KY401344.1
Tricholoma mongolicum voucher HMJAU:24943 18S ribosomal RNA gene, partial sequence; internal transcribed spacer 1, 5.8S ribosomal RNA gene, and internal transcribed spacer 2, complete sequence; and 28S ribosomal RNA gene, partial sequence	1024	1024	99%	0.0	95.64%	KC413946.1
Tricholoma mongolicum voucher HMJAU:24938 18S ribosomal RNA gene, partial sequence; internal transcribed spacer 1, 5.8S ribosomal RNA gene, and internal transcribed spacer 2, complete sequence; and 28S ribosomal RNA gene, partial sequence	1024	1024	99%	0.0	95.64%	KC413941.1
Tricholoma mongolicum voucher HMJAU:24944 18S ribosomal RNA gene, partial sequence; internal transcribed spacer 1, 5.8S ribosomal RNA gene, and internal transcribed spacer 2, complete sequence; and 28S ribosomal RNA gene, partial sequence	1022	1022	99%	0.0	95.77%	KC413947.1
Clitocybe cf. nuda voucher Mushroom Observer 329535 internal transcribed spacer 1, partial sequence; 5.8S ribosomal RNA gene and internal transcribed spacer 2, complete sequence; and large subunit ribosomal RNA gene, partial sequence	1018	1018	99%	0.0	95.48%	MT357078.1

Description	Max Score	Total Score	Query Cover	E value	Per. Ident	Accession
Leucocalocybe mongolica strain MCCJLAU2015C1 small subunit ribosomal RNA gene, partial sequence; internal transcribed spacer 1 and 5.8S ribosomal RNA gene, complete sequence; and internal transcribed spacer 2, partial sequence	1018	1018	98%	0.0	95.75%	KX641897.1
Leucocalocybe mongolica voucher HMAS 281506 internal transcribed spacer 1, partial sequence; 5.8S ribosomal RNA gene and internal transcribed spacer 2, complete sequence; and large subunit ribosomal RNA gene, partial sequence	1005	1005	97%	0.0	95.70%	MK966647.1
Lepista nuda strain dd08044 18S ribosomal RNA gene, partial sequence; internal transcribed spacer 1, 5.8S ribosomal RNA gene, and internal transcribed spacer 2, complete sequence; and 28S ribosomal RNA gene, partial sequence	1003	1003	99%	0.0	94.91%	FJ810156.1
Lepista nuda genes for 18S rRNA, ITS1, 5.8S rRNA, ITS2, 28S rRNA, partial and complete sequence, strain: LN13-1	1000	1000	99%	0.0	94.89%	LC144831.1
Lepista sordida voucher AH39056 18S ribosomal RNA gene, partial sequence; internal transcribed spacer 1, 5.8S ribosomal RNA gene, and internal transcribed spacer 2, complete sequence; and 28S ribosomal RNA gene, partial sequence	1000	1000	98%	0.0	95.27%	KJ681018.1
Lepista saeva voucher AH39129 18S ribosomal RNA gene, partial sequence; internal transcribed spacer 1, 5.8S ribosomal RNA gene, and internal transcribed spacer 2, complete sequence; and 28S ribosomal RNA gene, partial sequence	998	998	98%	0.0	95.13%	KJ681014.1
Clitocybe fragrans voucher 2148 18S ribosomal RNA gene, internal transcribed spacer 1, 5.8S ribosomal RNA gene, internal transcribed spacer 2, and 28S ribosomal RNA gene, region	998	998	99%	0.0	94.88%	JF907811.1
Lepista nuda genes for 18S rRNA, ITS1, 5.8S rRNA, ITS2, 28S rRNA, partial and complete sequence, clone: HS_1_ITS_060920_12	998	998	99%	0.0	94.75%	AB285100.1
Clitocybe sp. voucher S.D. Russell MycoMap 6981 small subunit ribosomal RNA gene, partial sequence; internal transcribed spacer 1, 5.8S ribosomal RNA gene, and internal transcribed spacer 2, complete sequence; and large subunit ribosomal RNA gene, partial sequence	996	996	99%	0.0	94.74%	MK532771.1
Lepista sp. AH39232 18S ribosomal RNA gene, partial sequence; internal transcribed spacer 1, 5.8S ribosomal RNA gene, and internal transcribed spacer 2, complete sequence; and 28S ribosomal RNA gene, partial sequence	996	996	98%	0.0	95.12%	KJ681017.1
Lepista nuda Ln16-1 genes for ITS1, 5.8S rRNA, ITS2, 28S rRNA, partial and complete sequence	994	994	99%	0.0	94.74%	LC370440.1
Lepista nuda genes for 18S rRNA, ITS1, 5.8S rRNA, ITS2, 28S rRNA, partial and complete sequence, strain: LN13-2	994	994	98%	0.0	94.87%	LC144832.1

Description	Max Score	Total Score	Query Cover	E value	Per. Ident	Accession
Lepista sordida voucher AH39148 18S ribosomal RNA gene, partial sequence; internal transcribed spacer 1, 5.8S ribosomal RNA gene, and internal transcribed spacer 2, complete sequence; and 28S ribosomal RNA gene, partial sequence	994	994	98%	0.0	95.11%	KJ681020.1
Lepista sordida voucher AH39147 18S ribosomal RNA gene, partial sequence; internal transcribed spacer 1, 5.8S ribosomal RNA gene, and internal transcribed spacer 2, complete sequence; and 28S ribosomal RNA gene, partial sequence	994	994	97%	0.0	95.25%	KJ681019.1
Clitocybe sp. 2 PA-2015 voucher AH42904 18S ribosomal RNA gene, partial sequence; internal transcribed spacer 1, 5.8S ribosomal RNA gene, and internal transcribed spacer 2, complete sequence; and 28S ribosomal RNA gene, partial sequence	994	994	99%	0.0	94.60%	KJ680982.1
Lepista nuda Ln17-6 genes for ITS1, 5.8S rRNA, ITS2, 28S rRNA, partial and complete sequence	992	992	99%	0.0	94.73%	LC370447.1
Uncultured fungus clone TSPF_32 18S ribosomal RNA gene, partial sequence; internal transcribed spacer 1, 5.8S ribosomal RNA gene, and internal transcribed spacer 2, complete sequence; and 28S ribosomal RNA gene, partial sequence	992	992	99%	0.0	94.60%	FJ213528.1
Uncultured fungus clone TSPF_1 18S ribosomal RNA gene, partial sequence; internal transcribed spacer 1, 5.8S ribosomal RNA gene, and internal transcribed spacer 2, complete sequence; and 28S ribosomal RNA gene, partial sequence	992	992	99%	0.0	94.60%	FJ213504.1
Lepista nuda genes for 18S rRNA, ITS1, 5.8S rRNA, ITS2, 28S rRNA, partial and complete sequence, clone: HS_1_ITS_060920_11	992	992	99%	0.0	94.60%	AB285106.1
Tricholoma mongolicum strain M5 internal transcribed spacer 1, partial sequence; 5.8S ribosomal RNA gene, complete sequence; and internal transcribed spacer 2, partial sequence	990	990	95%	0.0	95.79%	HQ446478.1
Tricholoma mongolicum strain M2 internal transcribed spacer 1, partial sequence; 5.8S ribosomal RNA gene, complete sequence; and internal transcribed spacer 2, partial sequence	990	990	95%	0.0	95.79%	HQ446476.1
Collybia tuberosa voucher MQ17019-QFB29527 small subunit ribosomal RNA gene, partial sequence; internal transcribed spacer 1, 5.8S ribosomal RNA gene, and internal transcribed spacer 2, complete sequence; and large subunit ribosomal RNA gene, partial sequence	989	989	99%	0.0	94.58%	MN992575.1
Collybia tuberosa voucher ANT217-QFB28689 small subunit ribosomal RNA gene, partial sequence; internal transcribed spacer 1, 5.8S ribosomal RNA gene, and internal transcribed spacer 2, complete sequence; and large subunit ribosomal RNA gene, partial sequence	989	989	99%	0.0	94.58%	MN992323.1

Description	Max Score	Total Score	Query Cover	E value	Per. Ident	Accession
Collybia tuberosa voucher ANT116-QFB28739 small subunit ribosomal RNA gene, partial sequence; internal transcribed spacer 1, 5.8S ribosomal RNA gene, and internal transcribed spacer 2, complete sequence; and large subunit ribosomal RNA gene, partial sequence	989	989	99%	0.0	94.58%	MN992322.1
Collybia tuberosa voucher MQ18R163-QFB30679 small subunit ribosomal RNA gene, partial sequence; internal transcribed spacer 1, 5.8S ribosomal RNA gene, and internal transcribed spacer 2, complete sequence; and large subunit ribosomal RNA gene, partial sequence	989	989	99%	0.0	94.58%	MN992127.1
Collybia tuberosa voucher MQ18R245-QFB30761 internal transcribed spacer 1, partial sequence; 5.8S ribosomal RNA gene and internal transcribed spacer 2, complete sequence; and large subunit ribosomal RNA gene, partial sequence	989	989	99%	0.0	94.57%	MN992126.1
Lepista saeva voucher AH39154 18S ribosomal RNA gene, partial sequence; internal transcribed spacer 1, 5.8S ribosomal RNA gene, and internal transcribed spacer 2, complete sequence; and 28S ribosomal RNA gene, partial sequence	989	989	98%	0.0	94.95%	KJ681015.1
Lepista saeva small subunit ribosomal RNA gene, partial sequence; internal transcribed spacer 1, 5.8S ribosomal RNA gene, and internal transcribed spacer 2, complete sequence; and large subunit ribosomal RNA gene, partial sequence	989	989	99%	0.0	94.71%	KJ137270.1
Uncultured fungus DNA sequence containing 18S rRNA gene, ITS1, 5.8S rRNA gene, ITS2 and 28S rRNA gene, isolate B0809	989	989	99%	0.0	94.71%	FM999669.1
Collybia tuberosa strain DAOM 191061 18S ribosomal RNA gene, partial sequence; internal transcribed spacer 1, 5.8S ribosomal RNA gene and internal transcribed spacer 2, complete sequence; and large subunit ribosomal RNA gene, partial sequence	989	989	99%	0.0	94.58%	AF274376.1
Collybia tuberosa strain Tennessee 7265 18S ribosomal RNA gene, partial sequence; internal transcribed spacer 1, 5.8S ribosomal RNA gene and internal transcribed spacer 2, complete sequence; and large subunit ribosomal RNA gene, partial sequence	989	989	99%	0.0	94.58%	AF065124.1
Clitocybe sp. 1 JL-2020 voucher MQ17063-QFB29571-HRL2439 small subunit ribosomal RNA gene, partial sequence; internal transcribed spacer 1, 5.8S ribosomal RNA gene, and internal transcribed spacer 2, complete sequence; and large subunit ribosomal RNA gene, partial sequence	987	987	99%	0.0	94.57%	MN992572.1
Clitocybe sp. voucher Mushroom Observer # 303033 small subunit ribosomal RNA gene, partial sequence; internal transcribed spacer 1, 5.8S ribosomal RNA gene, and internal transcribed spacer 2, complete sequence; and large subunit ribosomal RNA gene, partial sequence	987	987	99%	0.0	94.56%	MK607562.1
Lepista nuda Ln17-2 genes for ITS1, 5.8S rRNA, ITS2, 28S rRNA, partial and complete sequence	987	987	98%	0.0	94.84%	LC370443.1

Description	Max Score	Total Score	Query Cover	E value	Per. Ident	Accession
Lepista irina Li16-2 genes for ITS1, 5.8S rRNA, ITS2, 28S rRNA, partial and complete sequence	987	987	99%	0.0	94.70%	LC370439.1
Tricholoma mongolicum strain M7 internal transcribed spacer 1, partial sequence; 5.8S ribosomal RNA gene, complete sequence; and internal transcribed spacer 2, partial sequence	987	987	95%	0.0	95.77%	HQ446480.1
Collybia tuberosa isolate AFTOL-ID 557 18S ribosomal RNA gene, partial sequence; internal transcribed spacer 1, 5.8S ribosomal RNA gene, and internal transcribed spacer 2, complete sequence; and 25S ribosomal RNA gene, partial sequence	987	987	99%	0.0	94.57%	AY854072.1
Gymnopus sp. 1 JL-2020 voucher MQ17235-QFB29743 small subunit ribosomal RNA gene, partial sequence; internal transcribed spacer 1, 5.8S ribosomal RNA gene, and internal transcribed spacer 2, complete sequence; and large subunit ribosomal RNA gene, partial sequence	985	985	99%	0.0	94.56%	MN992594.1
Clitocybe odora voucher MQ18R044-QFB30127 small subunit ribosomal RNA gene, partial sequence; internal transcribed spacer 1, 5.8S ribosomal RNA gene, and internal transcribed spacer 2, complete sequence; and large subunit ribosomal RNA gene, partial sequence	985	985	99%	0.0	94.56%	MN992124.1
Clitocybe odora voucher 121 small subunit ribosomal RNA gene, partial sequence; internal transcribed spacer 1, 5.8S ribosomal RNA gene, and internal transcribed spacer 2, complete sequence; and large subunit ribosomal RNA gene, partial sequence	985	985	99%	0.0	94.56%	MH930123.1
Clitocybe sp. voucher 140_F06 small subunit ribosomal RNA gene, partial sequence; internal transcribed spacer 1 and 5.8S ribosomal RNA gene, complete sequence; and internal transcribed spacer 2, partial sequence	985	985	98%	0.0	94.83%	MK627492.1
Lepista irina strain CBS 551.87 small subunit ribosomal RNA gene, partial sequence; internal transcribed spacer 1, 5.8S ribosomal RNA gene, and internal transcribed spacer 2, complete sequence; and large subunit ribosomal RNA gene, partial sequence	985	985	99%	0.0	94.56%	MH862097.1
Lepista irina strain CBS 366.47 small subunit ribosomal RNA gene, partial sequence; internal transcribed spacer 1, 5.8S ribosomal RNA gene, and internal transcribed spacer 2, complete sequence; and large subunit ribosomal RNA gene, partial sequence	985	985	99%	0.0	94.56%	MH856287.1
Lepista saeva strain BAH08 (TENN) small subunit ribosomal RNA gene, partial sequence; internal transcribed spacer 1, 5.8S ribosomal RNA gene, and internal transcribed spacer 2, complete sequence; and large subunit ribosomal RNA gene, partial sequence	985	985	99%	0.0	94.56%	MF773596.1
Clitocybe sp. 1 PA-2015 voucher AH42909 18S ribosomal RNA gene, partial sequence; internal transcribed spacer 1, 5.8S ribosomal RNA gene, and internal transcribed spacer 2, complete sequence; and 28S ribosomal RNA gene, partial sequence	985	985	99%	0.0	94.56%	KJ680987.1

Description	Max Score	Total Score	Query Cover	E value	Per. Ident	Accession
Lepista nuda voucher CNF 1/7291 small subunit ribosomal RNA gene, partial sequence; internal transcribed spacer 1, 5.8S ribosomal RNA gene, and internal transcribed spacer 2, complete sequence; and large subunit ribosomal RNA gene, partial sequence	983	983	99%	0.0	94.30%	MK169235.1

Alignments

Lepista sordida culture-collection MFLUCC:13-0898 18S ribosomal RNA gene, partial sequence; internal transcribed spacer 1, 5.8S ribosomal RNA gene, and internal transcribed spacer 2, complete sequence; and 28S ribosomal RNA gene, partial sequence
Sequence ID: **KU877531.1** Length: 672 Number of Matches: 1
Range 1: 14 to 655

Score	Expect	Identities	Gaps	Strand	Frame
1173 bits(635)	0.0()	640/642(99%)	1/642(0%)	Plus/Minus	

Features:

```
Query  1    TCCTACCTGATTTGAGGTCAAAA-TGTCATAAATTTGTCCAAGTCAATGGACTGTTAGAA  59
Sbjct  655  TCCTACCTGATTTGAGGTCAAAAATGTCATAAATTTGTCCAAGTCAATGGACTGTTAGAA  596
Query  60   GCTGAACCCCATGTTAAAGCTGCTTCACAACCATGGCGTAGATAATTATCACACCAAAAG  119
Sbjct  595  GCTGAACCCCATGTTAAAGCTGCTTCACAACCATGGCGTAGATAATTATCACACCAAAAG  536
Query  120  CTGGTCCACAAAGGTTCCGCTAATGCATTTAAGAGGAGCCGACTTCTAGAGAAGCCCGCA  179
Sbjct  535  CTGGTCCACAAAGGTTCCGCTAATGCATTTAAGAGGAGCCGACTTCTAGAGAAGCCCGCA  476
Query  180  ATAACCTCCACATCCAAGCCAATCCAACTTGCAAAAGCTGAAAAGGTTGAGAATTTAATG  239
Sbjct  475  ATAACCTCCACATCCAAGCCAATCCAACTTGCAAAAGCTGAAAAGGTTGAGAATTTAATG  416
Query  240  ACACTCAAACAGGCATGCTCCTCGGAATACCAAGGAGCGCAAGGTGCGTTCAAAGATTCG  299
Sbjct  415  ACACTCAAACAGGCATGCTCCTCGGAATACCAAGGAGCGCAAGGTGCGTTCAAAGATTCG  356
Query  300  ATGATTCACTGAATTCTGCAATTCACATTACTTATCGCATTTCGCTGCGTTCTTCATCGA  359
Sbjct  355  ATGATTCACTGAATTCTGCAATTCACATTACTTATCGCATTTCGCTGCGTTCTTCATCGA  296
Query  360  TGCGAGAGCCAAGAGATCCGTTGTTGAAAGTTGTATTAATTTAAAGGCATAAAGCCCATT  419
Sbjct  295  TGCGAGAGCCAAGAGATCCGTTGTTGAAAGTTGTATTAATTTAAAGGCATAAAGCCCATT  236
Query  420  AATAACATTCTATTACATTCTTATGGGGTATAATAAAAACATAGACTTGAAACACAAGGA  479
Sbjct  235  AATAACATTCTATTACATTCTTATGGGGTATAATAAAAACATAGACTTGAAACACAAGGA  176
Query  480  AAGCCATGTTTGCACACAGCATTCCTCAAACCGAGTTTCCTCGAGAGTTGTTTCAAATCT  539
Sbjct  175  AAGCCATGTTTGCACACAGCATTCCTCAAACCGAGTTTCCTCGAGAGTTGTTTCAAATCT  116
Query  540  ACAAAAGGTGCACAGGTGGTAAAAATGGTGCTAGGCGTGCACATGCTCCAAAAAGCCAGC  599
Sbjct  115  ACAAAAGGTGCACAGGTGGTAAAAATGGCGCTAGGCGTGCACATGCTCCAAAAAGCCAGC  56
Query  600  ACAACCCAACCAAGTTTATTCAATAATGATCCTTCCGCAGGT  641
Sbjct  55   ACAACCCAACCAAGTTTATTCAATAATGATCCTTCCGCAGGT  14
```

Lepista sordida culture-collection MFLUCC:14-0769 18S ribosomal RNA gene, partial sequence; internal transcribed spacer 1, 5.8S ribosomal RNA gene, and internal transcribed spacer 2, complete sequence; and 28S ribosomal RNA gene, partial sequence
Sequence ID: **KU877530.1** Length: 672 Number of Matches: 1
Range 1: 14 to 655

Score	Expect	Identities	Gaps	Strand	Frame
1173 bits(635)	0.0()	640/642(99%)	1/642(0%)	Plus/Minus	

Features:

```
Query  1    TCCTACCTGATTTGAGGTCAAAA-TGTCATAAATTTGTCCAAGTCAATGGACTGTTAGAA  59
Sbjct  655  TCCTACCTGATTTGAGGTCAAAAATGTCATAAATTTGTCCAAGTCAATGGACTGTTAGAA  596
Query  60   GCTGAACCCCATGTTAAAGCTGCTTCACAACCATGGCGTAGATAATTATCACACCAAAAG  119
Sbjct  595  GCTGAACCCCATGTTAAAGCTGCTTCACAACCATGGCGTAGATAATTATCACACCAAAAG  536
Query  120  CTGGTCCACAAAGGTTCCGCTAATGCATTTAAGAGGAGCCGACTTCTAGAGAAGCCCGCA  179
Sbjct  535  CTGGTCCACAAAGGTTCCGCTAATGCATTTAAGAGGAGCCGACTTCTAGAGAAGCCCGCA  476
```

```
Query  180   ATAACCTCCACATCCAAGCCAATCCAACTTGCAAAAGCTGAAAAGGTTGAGAATTTAATG  239
Sbjct  475   ATAACCTCCACATCCAAGCCAATCCAACTTGCAAAAGCTGAAAAGGTTGAGAATTTAATG  416
Query  240   ACACTCAAACAGGCATGCTCCTCGGAATACCAAGGAGCGCAAGGTGCGTTCAAAGATTCG  299
Sbjct  415   ACACTCAAACAGGCATGCTCCTCGGAATACCAAGGAGCGCAAGGTGCGTTCAAAGATTCG  356
Query  300   ATGATTCACTGAATTCTGCAATTCACATTACTTATCGCATTTCGCTGCGTTCTTCATCGA  359
Sbjct  355   ATGATTCACTGAATTCTGCAATTCACATTACTTATCGCATTTCGCTGCGTTCTTCATCGA  296
Query  360   TGCGAGAGCCAAGAGATCCGTTGTTGAAAGTTGTATTAATTTAAAGGCATAAAGCCCATT  419
Sbjct  295   TGCGAGAGCCAAGAGATCCGTTGTTGAAAGTTGTATTAATTTAAAGGCATAAAGCCCATT  236
Query  420   AATAACATTCTATTACATTCTTATGGGGTATAATAAAAACATAGACTTGAAACACAAGGA  479
Sbjct  235   AATAACATTCTATTACATTCTTATGGGGTATAATAAAAACATAGACTTGAAACACAAGGA  176
Query  480   AAGCCATGTTTGCACACAGCATTCCTCAAACCGAGTTTCCTCGAGAGTTGTTTCAAATCT  539
Sbjct  175   AAGCCATGTTTGCACACAGCATTCCTCAAACCGAGTTTCCTCGAGAGTTGTTTCAAATCT  116
Query  540   ACAAAAGGTGCACAGGTGGTAAAAATGGTGCTAGGCGTGCACATGCTCCAAAAAGCCAGC  599
Sbjct  115   ACAAAAGGTGCACAGGTGGTAAAAATGGCGCTAGGCGTGCACATGCTCCAAAAAGCCAGC  56
Query  600   ACAACCCAACCAAGTTTATTCAATAATGATCCTTCCGCAGGT  641
Sbjct  55    ACAACCCAACCAAGTTTATTCAATAATGATCCTTCCGCAGGT  14
```

Lepista sordida culture-collection MFLUCC:12-0476 18S ribosomal RNA gene, partial sequence; internal transcribed spacer 1, 5.8S ribosomal RNA gene, and internal transcribed spacer 2, complete sequence; and 28S ribosomal RNA gene, partial sequence
Sequence ID: **KU877529.1** Length: 672 Number of Matches: 1
Range 1: 14 to 655

Score	Expect	Identities	Gaps	Strand	Frame
1173 bits(635)	0.0()	640/642(99%)	1/642(0%)	Plus/Minus	

Features:

```
Query  1     TCCTACCTGATTTGAGGTCAAAA-TGTCATAAATTTGTCCAAGTCAATGGACTGTTAGAA  59
Sbjct  655   TCCTACCTGATTTGAGGTCAAAAATGTCATAAATTTGTCCAAGTCAATGGACTGTTAGAA  596
Query  60    GCTGAACCCCATGTTAAAGCTGCTTCACAACCATGGCGTAGATAATTATCACACCAAAAG  119
Sbjct  595   GCTGAACCCCATGTTAAAGCTGCTTCACAACCATGGCGTAGATAATTATCACACCAAAAG  536
Query  120   CTGGTCCACAAAGGTTCCGCTAATGCATTTAAGAGGAGCCGACTTCTAGAGAAGCCCGCA  179
Sbjct  535   CTGGTCCACAAAGGTTCCGCTAATGCATTTAAGAGGAGCCGACTTCTAGAGAAGCCCGCA  476
Query  180   ATAACCTCCACATCCAAGCCAATCCAACTTGCAAAAGCTGAAAAGGTTGAGAATTTAATG  239
Sbjct  475   ATAACCTCCACATCCAAGCCAATCCAACTTGCAAAAGCTGAAAAGGTTGAGAATTTAATG  416
Query  240   ACACTCAAACAGGCATGCTCCTCGGAATACCAAGGAGCGCAAGGTGCGTTCAAAGATTCG  299
Sbjct  415   ACACTCAAACAGGCATGCTCCTCGGAATACCAAGGAGCGCAAGGTGCGTTCAAAGATTCG  356
Query  300   ATGATTCACTGAATTCTGCAATTCACATTACTTATCGCATTTCGCTGCGTTCTTCATCGA  359
Sbjct  355   ATGATTCACTGAATTCTGCAATTCACATTACTTATCGCATTTCGCTGCGTTCTTCATCGA  296
Query  360   TGCGAGAGCCAAGAGATCCGTTGTTGAAAGTTGTATTAATTTAAAGGCATAAAGCCCATT  419
Sbjct  295   TGCGAGAGCCAAGAGATCCGTTGTTGAAAGTTGTATTAATTTAAAGGCATAAAGCCCATT  236
Query  420   AATAACATTCTATTACATTCTTATGGGGTATAATAAAAACATAGACTTGAAACACAAGGA  479
Sbjct  235   AATAACATTCTATTACATTCTTATGGGGTATAATAAAAACATAGACTTGAAACACAAGGA  176
Query  480   AAGCCATGTTTGCACACAGCATTCCTCAAACCGAGTTTCCTCGAGAGTTGTTTCAAATCT  539
Sbjct  175   AAGCCATGTTTGCACACAGCATTCCTCAAACCGAGTTTCCTCGAGAGTTGTTTCAAATCT  116
Query  540   ACAAAAGGTGCACAGGTGGTAAAAATGGTGCTAGGCGTGCACATGCTCCAAAAAGCCAGC  599
Sbjct  115   ACAAAAGGTGCACAGGTGGTAAAAATGGCGCTAGGCGTGCACATGCTCCAAAAAGCCAGC  56
Query  600   ACAACCCAACCAAGTTTATTCAATAATGATCCTTCCGCAGGT  641
Sbjct  55    ACAACCCAACCAAGTTTATTCAATAATGATCCTTCCGCAGGT  14
```

Lepista sordida voucher TNS-F-61698 18S ribosomal RNA gene, partial sequence; internal transcribed spacer 1, 5.8S ribosomal RNA gene, and internal transcribed spacer 2, complete sequence; and 28S ribosomal RNA gene, partial sequence
Sequence ID: **KP293583.1** Length: 743 Number of Matches: 1
Range 1: 57 to 698

Score	Expect	Identities	Gaps	Strand	Frame
1173 bits(635)	0.0()	640/642(99%)	1/642(0%)	Plus/Minus	

Features:

```
Query  1    TCCTACCTGATTTGAGGTCAAAA-TGTCATAAATTTGTCCAAGTCAATGGACTGTTAGAA  59
Sbjct  698  TCCTACCTGATTTGAGGTCAAAAATGTCATAAATTTGTCCAAGTCAATGGACTGTTAGAA  639
Query  60   GCTGAACCCCATGTTAAAGCTGCTTCACAACCATGGCGTAGATAATTATCACACCAAAAG  119
Sbjct  638  GCTGAACCCCATGTTAAAGCTGCTTCACAACCATGGCGTAGATAATTATCACACCAAAAG  579
Query  120  CTGGTCCACAAAGGTTCCGCTAATGCATTTAAGAGGAGCCGACTTCTAGAGAAGCCCGCA  179
Sbjct  578  CTGGTCCACAAAGGTTCCGCTAATGCATTTAAGAGGAGCCGACTTCTAGAGAAGCCCGCA  519
Query  180  ATAACCTCCACATCCAAGCCAATCCAACTTGCAAAAGCTGAAAAGGTTGAGAATTTAATG  239
Sbjct  518  ATAACCTCCACATCCAAGCCAATCCAACTTGCAAAAGCTGAAAAGGTTGAGAATTTAATG  459
Query  240  ACACTCAAACAGGCATGCTCCTCGGAATACCAAGGAGCGCAAGGTGCGTTCAAAGATTCG  299
Sbjct  458  ACACTCAAACAGGCATGCTCCTCGGAATACCAAGGAGCGCAAGGTGCGTTCAAAGATTCG  399
Query  300  ATGATTCACTGAATTCTGCAATTCACATTACTTATCGCATTTCGCTGCGTTCTTCATCGA  359
Sbjct  398  ATGATTCACTGAATTCTGCAATTCACATTACTTATCGCATTTCGCTGCGTTCTTCATCGA  339
Query  360  TGCGAGAGCCAAGAGATCCGTTGTTGAAAGTTGTATTAATTTAAAGGCATAAAGCCCATT  419
Sbjct  338  TGCGAGAGCCAAGAGATCCGTTGTTGAAAGTTGTATTAATTTAAAGGCATAAAGCCCATT  279
Query  420  AATAACATTCTATTACATTCTTATGGGGTATAATAAAAACATAGACTTGAAACACAAGGA  479
Sbjct  278  AATAACATTCTATTACATTCTTATGGGGTATAATAAAAACATAGACTTGAAACACAAGGA  219
Query  480  AAGCCATGTTTGCACACAGCATTCCTCAAACCGAGTTTCCTCGAGAGTTGTTTCAAATCT  539
Sbjct  218  AAGCCATGTTTGCACACAGCATTCCTCAAACCGAGTTTCCTCGAGAGTTGTTTCAAATCT  159
Query  540  ACAAAAGGTGCACAGGTGGTAAAAATGGTGCTAGGCGTGCACATGCTCCAAAAAGCCAGC  599
Sbjct  158  ACAAAAGGTGCACAGGTGGTAAAAATGGCGCTAGGCGTGCACATGCTCCAAAAAGCCAGC  99
Query  600  ACAACCCAACCAAGTTTATTCAATAATGATCCTTCCGCAGGT  641
Sbjct  98   ACAACCCAACCAAGTTTATTCAATAATGATCCTTCCGCAGGT  57
```

Lepista sordida genomic DNA sequence contains ITS1, 5.8S rRNA gene, ITS2, strain EGDA2
Sequence ID: **LN827702.1** Length: 663 Number of Matches: 1
Range 1: 1 to 642

Score	Expect	Identities	Gaps	Strand	Frame
1173 bits(635)	0.0()	640/642(99%)	1/642(0%)	Plus/Minus	

Features:

```
Query  1    TCCTACCTGATTTGAGGTCAAAA-TGTCATAAATTTGTCCAAGTCAATGGACTGTTAGAA  59
Sbjct  642  TCCTACCTGATTTGAGGTCAAAAATGTCATAAATTTGTCCAAGTCAATGGACTGTTAGAA  583
Query  60   GCTGAACCCCATGTTAAAGCTGCTTCACAACCATGGCGTAGATAATTATCACACCAAAAG  119
Sbjct  582  GCTGAACCCCATGTTAAAGCTGCTTCACAACCATGGCGTAGATAATTATCACACCAAAAG  523
Query  120  CTGGTCCACAAAGGTTCCGCTAATGCATTTAAGAGGAGCCGACTTCTAGAGAAGCCCGCA  179
Sbjct  522  CTGGTCCACAAAGGTTCCGCTAATGCATTTAAGAGGAGCCGACTTCTAGAGAAGCCCGCA  463
Query  180  ATAACCTCCACATCCAAGCCAATCCAACTTGCAAAAGCTGAAAAGGTTGAGAATTTAATG  239
Sbjct  462  ATAACCTCCACATCCAAGCCAATCCAACTTGCAAAAGCTGAAAAGGTTGAGAATTTAATG  403
Query  240  ACACTCAAACAGGCATGCTCCTCGGAATACCAAGGAGCGCAAGGTGCGTTCAAAGATTCG  299
Sbjct  402  ACACTCAAACAGGCATGCTCCTCGGAATACCAAGGAGCGCAAGGTGCGTTCAAAGATTCG  343
Query  300  ATGATTCACTGAATTCTGCAATTCACATTACTTATCGCATTTCGCTGCGTTCTTCATCGA  359
Sbjct  342  ATGATTCACTGAATTCTGCAATTCACATTACTTATCGCATTTCGCTGCGTTCTTCATCGA  283
Query  360  TGCGAGAGCCAAGAGATCCGTTGTTGAAAGTTGTATTAATTTAAAGGCATAAAGCCCATT  419
Sbjct  282  TGCGAGAGCCAAGAGATCCGTTGTTGAAAGTTGTATTAATTTAAAGGCATAAAGCCCATT  223
Query  420  AATAACATTCTATTACATTCTTATGGGGTATAATAAAAACATAGACTTGAAACACAAGGA  479
Sbjct  222  AATAACATTCTATTACATTCTTATGGGGTATAATAAAAACATAGACTTGAAACACAAGGA  163
Query  480  AAGCCATGTTTGCACACAGCATTCCTCAAACCGAGTTTCCTCGAGAGTTGTTTCAAATCT  539
Sbjct  162  AAGCCATGTTTGCACACAGCATTCCTCAAACCGAGTTTCCTCGAGAGTTGTTTCAAATCT  103
Query  540  ACAAAAGGTGCACAGGTGGTAAAAATGGTGCTAGGCGTGCACATGCTCCAAAAAGCCAGC  599
Sbjct  102  ACAAAAGGTGCACAGGTGGTAAAAATGGCGCTAGGCGTGCACATGCTCCAAAAAGCCAGC  43
Query  600  ACAACCCAACCAAGTTTATTCAATAATGATCCTTCCGCAGGT  641
Sbjct  42   ACAACCCAACCAAGTTTATTCAATAATGATCCTTCCGCAGGT  1
```

BLAST is a registered trademark of the National Library of Medicine

Support center Mailing list YouTube

- National Library Of Medicine
- National Institutes Of Health
- U.S. Department of Health & Human Services
- USA.gov

NCBI

National Center for Biotechnology Information, U.S. National Library of Medicine 8600 Rockville Pike, Bethesda MD, 20894 USA
Policies and Guidelines | Contact

COVID-19 is an emerging, rapidly evolving situation.
Get the latest public health information from CDC: https://www.coronavirus.gov .
Get the latest research from NIH: https://www.nih.gov/coronavirus.
Find NCBI SARS-CoV-2 literature, sequence, and clinical content: https://www.ncbi.nlm.nih.gov/sars-cov-2/.
