# Supplementary material for: The physiological response of Ectomycorrhizal fungus Lepista sordida to Cd and Cu stress
Source: PeerJ. 2021 Apr 16;9:e11115. doi: 10.7717/peerj.11115 (PMC8054734; doi:10.7717/peerj.11115)
Supplement: Supplemental Information 2 [file peerj-09-11115-s002.docx]

<https://www.ncbi.nlm.nih.gov/nuccore/MT645231>


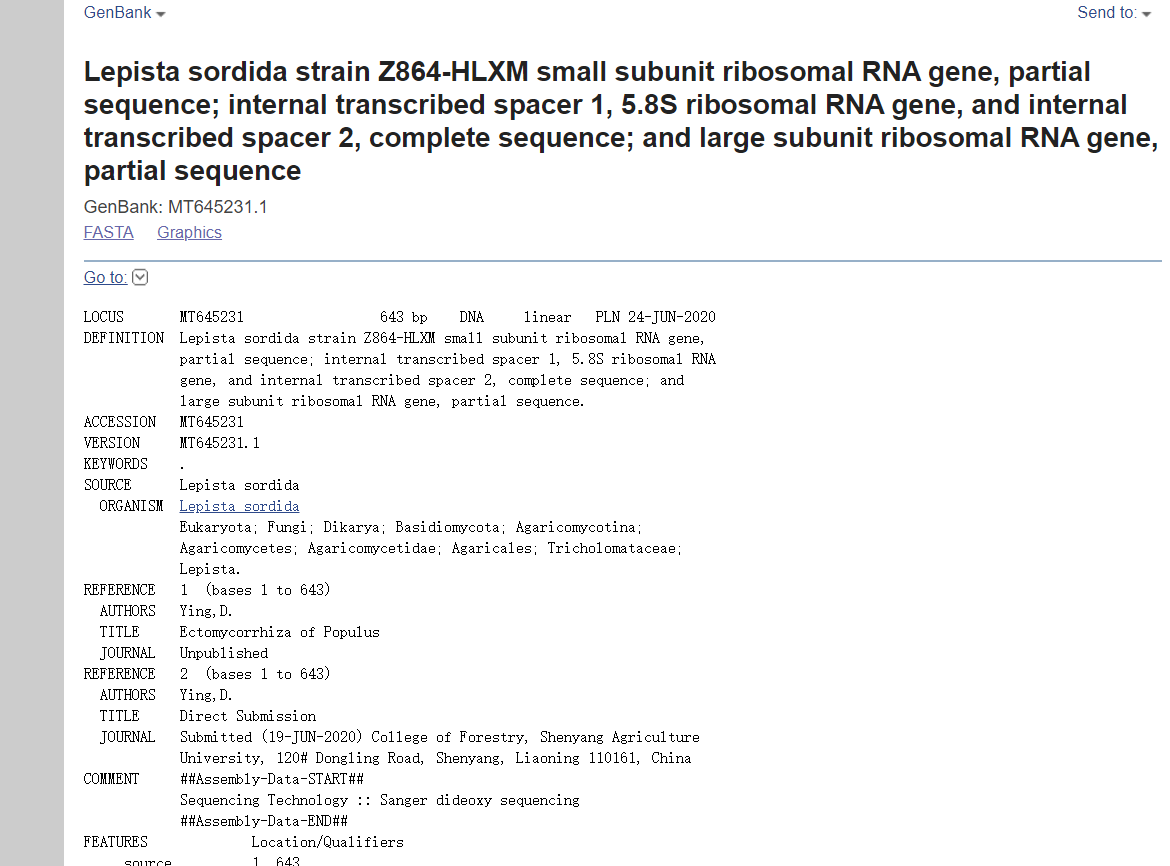


The sequence was submitted to GenBank to obtain the gene login number MT645231.
