# Supplementary material for: The physiological response of Ectomycorrhizal fungus Lepista sordida to Cd and Cu stress
Source: PeerJ. 2021 Apr 16;9:e11115. doi: 10.7717/peerj.11115 (PMC8054734; doi:10.7717/peerj.11115)
Supplement: Supplemental Information 3 [file peerj-09-11115-s003.docx]

TcCTACCTGATTTGAGGTCAAAATGTCATAAATTTGTCCAAGTCAATGGACTGTTAGAAGCTGAACCCCATGTTAAAGCT

GCTTCACAACCATGGCGTAGATAATTATCACACCAAAAGCTGGTCCACAAAGGTTCCGCTAATGCATTTAAGAGGAGCCG

ACTTCTAGAGAAGCCCGCAATAACCTCCACATCCAAGCCAATCCAACTTGCAAAAGCTGAAAAGGTTGAGAATTTAATGA

CACTCAAACAGGCATGCTCCTCGGAATACCAAGGAGCGCAAGGTGCGTTCAAAGATTCGATGATTCACTGAATTCTGCAA

TTCACATTACTTATCGCATTTCGCTGCGTTCTTCATCGATGCGAGAGCCAAGAGATCCGTTGTTGAAAGTTGTATTAATT

TAAAGGCATAAAGCCCATTAATAACATTCTATTACATTCTTATGGGGTATAATAAAAACATAGACTTGAAACACAAGGAA

AGCCATGTTTGCACACAGCATTCCTCAAACCGAGTTTCCTCGAGAGTTGTTTCAAATCTACAAAAGGTGCACAGGTGGTA

AAAATGGTGCTAGGCGTGCACATGCTCCAAAAAGCCAGCACAACCCAACCAAGTTTATTCAATAATGATCCTTCCGCAGG

TAC
